# Crop water requirement and irrigation scheduling under climate change scenario, and optimal cropland allocation in lower kulfo catchment

**DOI:** 10.1016/j.heliyon.2024.e31332

**Published:** 2024-05-16

**Authors:** Birara Gebeyhu Reta, Samuel Dagalo Hatiye, Mekuanent Muluneh Finsa

**Affiliations:** a Faculty of Water Resources and Irrigation Engineering, Arba Minch Water Technology Institute, Arba Minch University, Arba Minch, Ethiopia; b Water Resources Research Centre, Arba Minch Water Technology Institute, Arba Minch University, Arba Minch, Ethiopia

**Keywords:** GAMS code, CropWat, Climate change, Cropland allocation

## Abstract

Crop water requirement and irrigation scheduling in Lower Kulfo Catchment of southern Ethiopia have not assessed under climate change scenarios, and the allocation of crop land also not optimal that signifcantly challenges to crop productivity.Therefore, this study was conducted to evaluate the effects of climate change on future crop water requirements, and irrigation scheduling, and to allocate cropland optimally. Bias of projected precipitation and temperature were corrected by utilizing Climate Model data with the hydrologic modeling tool (CMhyd). Alongside, crop water requirements and irrigation scheduling were assessed using Crop Water Assessment Tool. After estimating crop water requirement, crop land were allocated optimally using General Algebraic Modeling System programming with non-negativity constraints (scenario 1), and non-negativity constraints based on farmers adaptation (scenario 2). Average reference evapotranspiration from 2030 to 2050 and 2060 to 2080 was increased by 11.9 %, and 16.2 %, respectively compared with the reference period (2010–2022). The total seasonal crop water requirements were 4,529 mm, 4866.7 mm, and 5272.2 mm under 2010 to 2022, 2030 to 2050, and 2060 to 2080 climate change scenarios, respectively. The meean irrigation interval in 2010–2022, 2030 to 2050, and 2060 to 2080 climate change scenarios were 8 days, 7 days, and 5 days, respectively. This irrigation interval was decreased by 14 % (2030–2050), and 34 % (2060–2080) compared with the reference period. In 2030 to 2050 and 2026 to 2080 climate change scenarios, the required irrigation water at the inlet of main canal increased by 6.8 %, and 18 %, respectively. The optimal allocated area for tomato (60.4 %), maize (20.8 %), and watermelon (18.8 %) in scenario 1 with net benefit of 1.47*108 Ethiopian Birr. The allocated areas in scenario 2 were (48 %) for maize, (31.6 %) for tomato, and (20.4 %) for watermelon with 1.34*10^8^ Ethiopian Birr net benefit it was reduced by 19.1 % compared with the net benefit in scenario 1. Fruit crops alone may not suffice for local food needs and to address this, small farmers should grow maize, tomato, and watermelon. This research aids policymakers in encouraging climate-resilient agriculture and improving small-scale farmers' awareness through conducting workshops and training.

## 1 Introduction

### 1.1 Background

Agriculture plays an important role in driving economic growth within the Ethiopian economy and it covers 40 % of gross domestic product [1]. Irrigation agriculture in dry or semi-dry environments is used to sustain agricultural productivity when available rainfall is insufficient [2]. Effective irrigation water management across the water conveyance system and demand-based irrigation scheduling are the basic activities to improve the productivity of irrigation schemes [3]. Crop water requirement is the amount of water equal to what is lost from the cropped field by evapotranspiration [4]. Soil type, climate change, topographical location, and crop type are highly affect the quantity of crop water requirement [5]. Climate change denotes significant and enduring shifts in Earth's climate, driven by human activities due to emitting of greenhouse gases like CO_2_, CH_4_, and N_2_O, and altering temperature, precipitation, and wind patterns [6]. The estimation of climate change's impact on crop water requirement is used to suggest possible mitigation measures for sustainable water resources development [7].

The Coupled Model Intercomparison Project (CMIP) projected climate driving model is organized by the World Climate Research Program (WCRP) it produces ensembles of Earth System Model (ESM) projected future climate conditions based on different CO_2_ emission scenarios [8]. In comparison to CMIP5, CMIP6 is the most recent phase with high spatial and temporal resolutions that offer more intricate representation of climate processes [9]. To forecasting future climate change scenarios, CMIP6 (SSP585) demonstrates better performance when contrasted with CMIP5 (SSP585) [10]. Crop Water Assessment Tool (CropWat model) is used to estimate crop water requirement and irrigation scheduling using historical or future projected temperature and precipitation [11]. Climate Model data with the hydrologic modeling tool (CMhyd) is used to utilize bias correction between historical and projected climate [12] that is used as input for the CropWat model.

Proper irrigation scheduling under climate change scenarios is used to increase yields and manage the amount, and frequency of irrigation [13] and it also gives a direction to adapted climate resilience agriculture. Optimizing agricultural land utilization is important to satisfy household food security by providing most economical crops for the specific area [14]. This crop optimization model contains objective function, decision variables [15], and constraints that depending on nature of the problems [16]. Crop allocation model can consider water and land availability [17] and the aims of optimization also to maximize agricultural net benefit per unit of water or land [18]. General Algebraic Modeling systems (GAMS) code programming is the best tool to allocate agricultural land under different cropping patterns and this allocation strategy considers water, land, crop, and economic constraints [19].

During dry season, there was water conflict among the water users in Lower Kulfo Catchment due to the scarcity of irrigation water and the amount of irrigation demand showed an increasing trend as observed during problem investigation that may be due to climate change. Absence of estimated crop water requirement and lack of proper irrigation scheduling practices in the Lower Kulfo Catchment was significantly disturbed water management and distribution. Addition to this, rainfall variability and shifting of wet season also the major problems in the lower Kulfo catchment that hinder rainfed/irrigated agriculture in the area. Stream flow of the Kulfo River will be decreased by 2.99 % in the 2050s and 5.28 % in the 2080s due to climate change impact [20]. But there was no any conducted research in Lower Kulfo Catchment to evaluate the impacts of climate change on crop water requirement and irrigation scheduling. Both land and water productivity of crops Arba Minch irrigation in the Lower Kulfo Catchment are low [21] and that may be lack of knowledge about user-friendly crop optimization tools for identifying economical crops to the area. Traditional allocation of cropland was adopted by irrigation users in the lower Kulfo catchment that was due to lack of understanding regarding affordable crop optimization programming like GAMS code. Poor optimal cropland allocation under multiple cropping patterns undermines the effectiveness of an irrigation scheme, leading to reduced crop yields, increased operational costs, market value fluctuations, and environmental degradation [22].

These agricultural-related problems can be solved by developing reasonable irrigation scheduling and estimating crop water requirements under climate change scenarios, and optimal cropland allocation is also used to identify the most economical crops in the area. Therefore, this study was conducted to estimate crop water requirement and irrigation scheduling for the worst dry season under three climate change scenarios and to allocate cropland under multiple crop systems in the lower Kulfo catchment using GAMS code programming. The significance of this research lies in its potential to address critical challenges in the lower Kulfo catchment, offering solutions for optimizing water resources, improving agricultural productivity, and fostering sustainable practices in the face of climate change.

## 2 Material and methods

### 2.1 Description of the study area

**Fig. 1:**
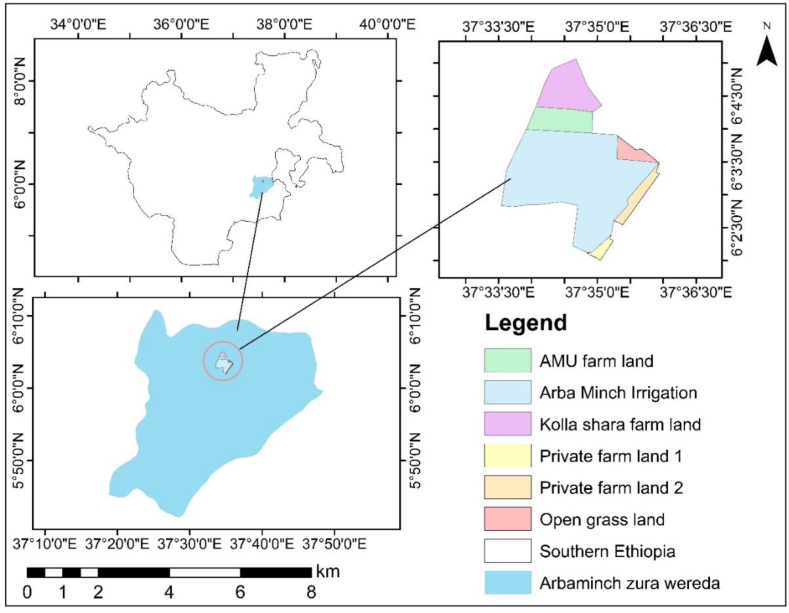
Location of study area.

Lower Kulfo catchment was located between 6 ° 2′ 0″ and 6 ° 5′ 0″ North latitude and 37 ° 33′0″ and 37 ° 36′0″ East longitudes of Southern Ethiopia (Fig. 1). Elevation of thestudy area was varied from 1200 to 1203.8 m above the mean sea. The Lower Kulfo catchment was located near Arba Minch town, running alongside the main road connecting Arba Minch to Mirab Abaya and Wolayita Sodo and this location holds significant importance for the efficient transportation of fruit production to the market. Arba Minch irrigation, Arba Minch University (AMU) farmland, smallhold farmer in the Kola Shara district, and private farmland near the Arba Minch airport were included in the study area. The irrigated area of the Arba Minch irrigation scheme, Arba Minch University farm, Kolla shara farmland, private farmland 1, and private farmland 2 in Lower Kulfo Catchment were 835.22ha, 109.17ha, 160.23ha, 18.44ha, and 52.76ha, respectively, and the total irrigable land was 1175.82ha (Fig. 1). The water source of the Lower Kulfo Catchment was Kulfo River and the annual minimum, and maximum flow of the River were 2.35 m^3^/s and 50.73 m^3^/s, respectively.

### 2.2 Data collection

Market survey was conducted to collect local price of the dominant crop in the study area and the survey included both sellers and buyers in the market which helps to identify crops that are used by people since only crop prices are not used to justify whether the crop is profitable or not. Field observation was conducted around the Lower Kulfo Catchment to investigate the most practical agricultural crop and this field observation also used to understand the agronomic performance of crops in the area. Both quantitative and qualitative data regarding crop production practices such as land size, costs of crop production, existing farming practices, and productivity of crops per hectare were collected through household survey questionnaires, key informant interviews, and focus group discussions. This data was used to estimate the costs and revenues of crop production per hectare for each crop. Based on the [23], simplified formula, the sample size for household interviews in Kolla shara Kebele was calculated as described in Eq (1).

(1) $$n=\frac{N}{1+N\left(e\right)ˆ2}$$

where n and N are sample size and total population size respectively, and e is expected error (5 %) at a 95 % confidence level. The total population and available agricultural land were collected from Arba Minch Zuria Woreda office. The total irrigated land in Kolla Shara Kebele that will be irrigated with the Kulfo River was estimated with ArcGIS software after collecting of ground control point (Table 1).

**Table 1:** population and sample size to conduct the household interview in Kolla share.

Total population	10,794
Number of farmers	886
Available agricultural land (ha)	1974
Probable area irrigated by Kulfo River (ha)	160.2
Number of farmers under irrigated land (N)	72
Expected error (e)	5 % @95 % confidence level
Sample size to conduct a household interview	61

### 2.3 Soil physiochemical analysis

Methods of soil sampling were composite techniques and the maximum sampling depth is 0.9 m. Soil texture was evaluateing using a hydrometer test and bulk density also evaluated by dividing dry mass of soil sample by volume of after drying in oven dry at 105 for 24 h. Soil chemical properties such as soil organic matter and electric conductivity also estimated laboratory that used to justify present status of soil fertility. Soil field capacity and permanent wilting point were estimated by using pressure plate apparatus in the laboratory. Based on [24], infiltration characteristics of soil were determined by using a double-ring infiltrometer. These estimated soil physical properties were used as input for CropWat model to estimate crop water requirement and irrigation scheduling.

### 2.4 Climate change analysis

Based on [25], Climate Model Intercomparison Project 6 (CMIP6) has good performance compared with Phase 3 (CMIP3) and Phase five (CMIP5) in predicting future climate trends. As a result, the future temperature and precipitation of the current study was derived from the sixth phase of the Climate Model Intercomparison Project 6 (CMIP6). To utilized bias correction, historical temperature and precipitation were collected from the Arba Minch meteorological station. Temperature, and precipitation network Common Data Form (netCDF) files were extracted using coordinate and elevation of Arba Minch meteorological station. The Climate Model data for hydrologic modeling tool (CMhyd) was employed for bias correction of rainfall and temperature and based on [26], bias correction was addressed by Eqs (2) and (3).

(2) $$P^{*}=aP^{b}$$

where p* is the bias-corrected rainfall, P is the uncorrected rainfall amount, and a & b are the power regression factors.

(3) $$T^{*}=T_{obs}+\left(\frac{\delta \left({\bar{\;T}}_{obs}\right)}{\delta {\;(T}_{rcm})}\right)*\left(T_{rcm}-{\bar{T}}_{obs}\;\right)+\left({\bar{T}}_{obs}-{\bar{T}}_{rcm}\right)$$

T*, Tobs, Trcm, ${\bar{T}}_{obs}$, ${\bar{T}}_{rcm}$ and δ were stand for bias-corrected temperature, observed temperature, uncorrected projected temperature, mean of observed temperature, mean of uncorrected projected temperature, and standard deviation of observed or projected temperature, respectively.

where P* is the bias-corrected rainfall amount; P is the

uncorrected rainfall amount; a and b are factors.

### 2.5 CropWat model

Crop water assessment tool (CropWat) software is a computer program it was used estimate crop water requirements and irrigation scheduling using soil, climate, and crop data as input. Based on [27], reference evapotranspiration (ETo), crop water requirement (CWR), effective rainfall (Pe), irrigation water requirement (IWR), and irrigation interval (i) were evaluated through CropWat under different climate change scenarios. This climate scenario was 2010–2022 (reference), 2030 to 2050, and 2060–2080 and this future climate change was only temperature and rainfall. Projected solar radiation, humidity, wind speed and sunshine hours are not available in the Intercomparison Project 6 (CMIP6) model as result historical value of reference period was used to estimate reference evapotranspiration in all climate change scenarios. The driest season of study area was from January to April as a result, reference evapotranspiration, crop water requirement, and irrigation scheduling were evaluated for this worst condition in all climate change scenarios.

Crop coefficients of each crop for initial, mid, and late stages were collected from irrigation and drainage manual paper number 56 (FAO 56) as presented in Table 2 but crop coefficients of teff were not found in the FAO paper. Length of crop growing stage, root depth, yield reduction factor, allowable management depletion, and planting and harvesting date of crops were also collected from FAO irrigation and drainage paper. The crop coefficients of teff in central Rift Valley Lake Basin, Ethiopia were 0.46 (initial stage), 0.88 (development stage), 1.03 (mid-stage), and 0.57 (late stage) [28]. Reference evapotranspartion, crop water requirement, effective rainfall, irrigation requirement, and irrigation interval were evaluated by using Eq (4), Eq (5), Eq (6)/7, Eq (8) and Eq (9), respectively.

(4) $${ET}_{o}=\frac{0.408\Delta \left(R_{n}+G\right)+r*\frac{900}{T+273}U_{2}\left(e_{s}-e_{a}\right)}{\Delta +r\left(1+0.34U_{2}\right)}$$

(5) $$\text{CWR}=\text{kc}*{ET}_{o}$$

(6) $$\text{Pe}=\frac{\left(p*\left(125-0.2*3*p\right)\right)}{125}$$

;ifp≤250/3

(7) $$\text{Pe}=\frac{250}{3}+0.1p$$

;ifp>250/3

(8) IWR=CWR−Pe

(9) $$i=\frac{d}{CWR}$$

where; R_n_ = net radiation at the crop surface (MJ m-2 day-1), G = Soil heat flux density (MJ m-2 day-1), T = Mean daily air temperature at 2 m height (oc), U_2_=Wind speed at 2 m height (ms-1), e_s_ = Saturation vapour pressure (kPa), e_a_ = actual vapour pressure (kpa), (e_s_ -e_a_) = Saturated vapour pressure deficit, (kpa), Δ = slope vapour pressure curve (kPa oc-1) and r = psychrometric constant (kPa oc-1), Kc = crop coefficients (−), P is the total rainfall (mm), d is net irrigation depth (mm), and CWR is daily crop water requirement (mm/day).

**Table 2:** Crop coefficients as a function of crop type and crop growth stage [27].

Crop/Growth stage	Initial stage	Mid-stage	Late-stage
Wheat	0.3	1.15	0.25-0.4 (0.325)
Maize	0.3	1.2	0.6
Watermelon	0.4	1	0.75
Pepper	0.6	1.05	0.9
Onion	0.7	1.05	0.75
Banana	0.6	1.1	1.05
Tomato	0.15	1.1	0.6-0.8 (0.7)

### 2.6 GAMS code

General Algebraic Modeling System (GAMS) code was used to solve mixed-integer, linear, and nonlinear optimization problems [29]. Objective function of this research was to maximize the net profits of crop production in the catchment and it was develop based on [30] as described in Eq (10).

(10) $$\text{Maximize}\;Z=\sum_{i=1}^{n}\left(P_{i}Y_{i}X_{i}-C_{i}X_{i}\right)\;$$

where; P_i_, Y_i_, X_i_, and C_i_ were price for the crop “i” (birr/ton), the yield of the crop “i” (ton/ha), allocated area for crop “i” (ha) and production cost for the crop “i” (birr/ha), respectively.

Total production cost was including labor, fertilizer, pesticides, and insecticides cost and total irrigated land, water availability, expected outcome, total production cost and non-negativity were constraints of the objective function. Lengths of crop development stages for various planting periods were collected from Ref. [27] and crop planting was start in January according to the guidelines outlined in the FAO Irrigation and Drainage paper and only permanent banana crops persist year-round. Onions can be harvested by the end of March, while wheat crops require five months from the time of planting and irrigation will be stope after four months except perennial banana crop.

#### 2.6.1 Total land availability constraint

The sum of allocated areas for each crop will not exceed the total available land (At) as describe in Eq (11).

(11) $$X_{1}+X_{2}+X_{3}+X_{4}+X_{5}+X_{6}+X_{7}+X_{8}\leq A_{t}$$

where X_1_, X_2_, X_3_, X_4_, X_5_, X_6_, X_7_, and X_8_ are allocated areas for crop onion, maize, watermelon, pepper, wheat, banana, Teff, and tomato, respectively (ha) and A_t_ = total irrigated land (ha).

#### 2.6.2 Water availability constraint

The multiple product of irrigated land (ha) and gross irrigation depth (mm) was used to calculate the total volume of irrigation water. This value to be less than or equal to the seasonal minimum amount of water that could be obtained from the sources. Crop water requirement and effective rainfall under the reference period (2010–2022) was used to develop the Equation of water availability constraint and crop water requirement of banana after four months not included (Eq (12)).

(12) $$\sum_{n=1}^{\infty }\left({CWR}_{i}-\text{Peff}\right)X_{i}\leq V_{\min}\;$$

where; CWR = crop water requirement for the crop “i” (m), Peff = effective rainfall (m), V_min_ = annual minimum volume of water supply (ha-m).

#### 2.6.3 Expected total yield constraints

The expected crop yield represents the highest achievable productivity of a crop per hectare when soil quality is optimal, irrigation is managed effectively, and there is sufficient rainfall or irrigation water available. This expected maximum productivity data is sourced from the Irrigation and Drainage Paper Manual Number 33 (FAO 33). The primary aim of this research is to maximize the overall expected yield from the crops (Eq (13)).

(13) $$\sum_{i=1}^{n}\text{XIYI}\geq \sum_{i=1}^{n}\text{TYc}$$

where Y_I_ is the average expected land productivity for each crop (ton/ha) and TY_c_ = Expected total yield from all crops (ton).

#### 2.6.4 Production cost constraint

The sum of the production costs for each crop should not exceed with actual total production cost as describe in Eq (14).

(14) $$\sum_{i=1}^{n}\;X_{I}{PC}_{I}\leq \sum_{i=1}^{n}\text{TPC}$$

where; PC_i_ and TPC were crop production cost for crop I (Birr/ha) and total production cost (Birr) respectively.

Non-negativity constraints of optimization were had two scenarios such as allocated land for each crop was non-negativity (scenario 1) and the remaining non-negativity constraints also depend on small-hold farmer practices (scenario 2). The remaining constraints (such as land, water, expected yield, and production cost) were common in both scenarios.

#### 2.6.5 Non-negativity constraints (scenario 1)

The allocated area for each crop were considered as non-negativity during the GAMS code optimization (scenario 1).

X1 ≥ 0, X2 ≥ 0, X3 ≥ 0, X4 ≥ 0, X5>=………………………………………………………………………….X8 ≥ 0

#### 2.6.6 Non-negativity constraints based on farmers' adaptation (scenario 2)

Based on the household interview, the minimum area that covered by the maize crop under scenario 2 optimization was 564.9ha (48 % of total area). Because only fruit and vegetable production will not cover food consumption in Lower Kulfo Catchment due to that smallhold farmers allocate more land for maize. The other optimization also evaluated under 48 % area covered by maize crop in ordered to satisfy household food security in the area (scenario 2).

X_1_>=0, X_2_>=564.9ha, X_3_>=0, X_4_>=0, X_5_>=………………………………………..……X_8_>=0

## 3 Result and discussion

### 3.1 Soil physiochemical properties

Average soil texture was clay and the mean value of soil bulk density, soil organic matter, electric conductivity, field capacity, permanent wilting point, and total available water were 1.32 gm/cm^3^, 0.87 %, 0.16 ds/m, 38.3 %, 25.9 %, and 124 mm/m, respectively (Table 3). Based on [27], the maximum total available water of the clay soil varied from 110 to 160 mm/m, and the value of the current study was also found with the recommended value that was 127 mm/m. The soil was suitable for agricultural practices to be uncompacted with a bulk density of less or equal to 1.63 gm/cm^3^ [31]. Therefore, the bulk density of the current study was found with the recommended value which means Lower Kulfo Catchment was too suitable for agriculture practices with normal soil compactness status. Volumetric field capacity and permanent wilting point of clay soil was found 40 % and 25 % [32], 21.82 % & 35.03 % [33] and 21.% & 44.8 % [34], respectively, and value of current studies found between the recommended value (Table 3). According to Ref. [35] the recommended value soil organic matter on agricultural soils is between 3 % and 6 %. But value of current study was below the recommended value that indicates agricultural lands of current study need additional input like manure and mulching material to increase soil organic matter.Where, BD, SOM, ECe, FC, and PWP are bulk density, soil organic matter, extract electric conductivity, field capacity, and permanent wilting point of soil.

**Table 3:** Soil physiochemical properties.

Depth (cm)	% clay	% Silt	% Sand	Texture class	BD (gm/cm^3^)	SOM (%)	ECe (ds/m)	FC (%vol)	PWP (%vol)
0–30	41.8	30.9	27.3	Clay	1.28	0.90	0.19	38.1	25.5
30–60	42.5	22.2	35.4	Clay	1.31	0.88	0.15	38.7	25.6
60–90	43.7	21.2	35.1	Clay	1.36	0.83	0.13	38.3	26.7
**Mean**	**42.7**	**24.8**	**32.6**	**Clay**	**1.32**	**0.87**	**0.16**	**38.3**	**25.9**

This obtained average maximum and basic infiltration rate of soil was 1.40 and 0.08mmmin^-1^, respectively. The basic infiltration rate of clay and heavy clay soil varies from 2 to 5mmhr^−1^ [36] and that basic infiltration rate of soil was used as input for the CropWat model. The approximated basic soil infiltration rate assists in determining the optimal rate at which water should be applied to the soil, preventing issues such as runoff or waterlogging [37].

### 3.2 Crop pattern in lower kulfo catchment

**Fig. 2:**
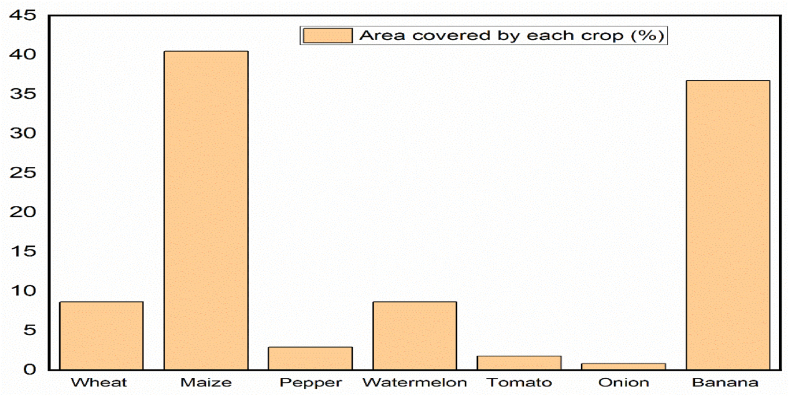
Cropping pattern in Arba Minch irrigation scheme.

Percentage of the area covered by each crop type was collected from Arba Minch irrigation scheme office on similar irrigation season and some amount of command area was not covered with the crop as presented in Fig. 2. Within the irrigated land of the Arba Minch irrigation scheme, maize and banana were the predominant crops.

The private enterprise in Lower Kulfo catchment only planted banana crops and the covering area was 71.1ha. The farm size of each farmer in Kolla Shara was varied from 0.25 to 3 ha, with an average size of 1.625ha. The farm sizes were categorized into four groups, as detailed in Table 4. Understanding the farm size of smallholder farmers is fundamental for planning, implementing, and managing irrigation systems effectively [38]. Therefore, this estimated agricultural land per farmer is important to fix the amount of irrigation water delivery at the time of irrigation and it was also important to manage the duration of irrigation.

**Table 4:** Ranges of farm size and numbers of farmers in Kolla Shara (Field survey).

s/no	Range of farm size (ha)	Numbers of farmers	Farm size in percent
**1**	0.25-0.5	10	16.39
**2**	0.5–1	19	31.15
**3**	1.0–2	14	22.95
**4**	>2	18	29.51

**Fig. 3:**
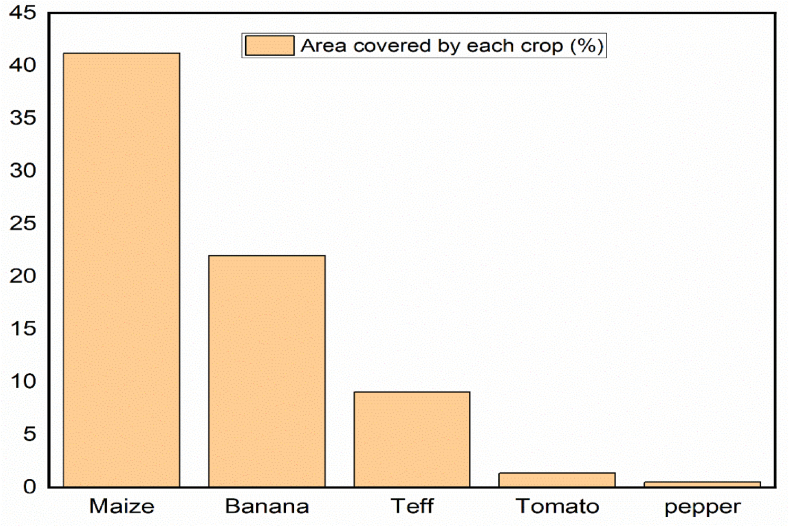
Percentages of the area covered by corresponding crop type in Kolla Shara kebele.

From household interview, the largest percentage of the area in Kolla Shara was covered by maize crops, and the second dominant crop also banana (Fig. 3) that have similar trends with the Arba Minch irrigation scheme. Pepper also covers with small area compared with other crop that show farmers in the area highly practices cereals crop agriculture like maize.

### 3.3 Climate change impact

#### 3.3.1 On temperature and rainfall

**Fig. 4:**
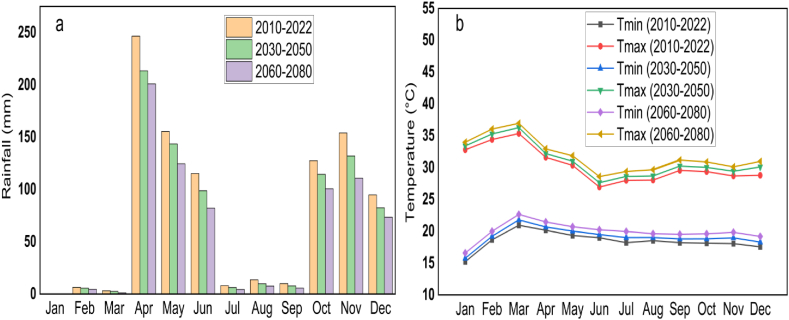
Monthly rainfall (a) and temperature (b) under climate change scenarios.

The average minimum (Tmin), and maximum temperature (Tmax) of the study area for the reference period was varied from 16 to 20 °C and 27–30 °C, respectively (Fig. 4a). This climate condition shows that irrigation is critically needed in the Lower Kulfo Catchment from January to March and from July to September. However, the period from January to March was the most severe dry season conditions as result the effects of climate change on crop water requirement and irrigation scheduling was assessed specifically for this irrigation season. Crop allocation was also evaluated for the worst dry season because analysis for the second season may not be significant because this season was slightly rainy compared with the worst dry season (January to March). The average annual rainfall and potential evapotranspiration of the Lower Kulfo Catchment were 904 mm and 1533 mm, respectively. Based on [23], the climate classification of the study was found under dry sub-humid climate zone that includes arid and semi-arid regions. The area also has a bimodal rainy season that was from April to Jun and from September to December. The minimum rainfall was observed in January and the rainy season was started on March 23 (Fig. 4a). The mean monthly temperature was increased by 3 %, and 6 % in the of 2030–2050 and 2060–2080 climate change scenarios, respectively compared with the reference scenario (2010–2022). Maximum temperatures was found in March across all scenarios, indicating an unprecedented rise in monthly temperatures. The monthly available rainfall also decreased by 15 % and 30.4 %, respectively, under the mentioned climate change scenarios and the maximum rainfall reduction was observed under the month of August (2030–2050) and March (2060–2080) (Fig. 4b). Effective water conservation strategies, such as rainwater harvesting, efficient irrigation practices, and sustainable water management, should be implemented to minimize climate change risk [39]. Additionally, promoting straw mulching [40] and deficit irrigation [41], afforestation and adopting climate-resilient crops [42] can enhance water retention and reduce vulnerability to climate change.

#### 3.3.2 On reference evapotranspiration

**Fig. 5:**
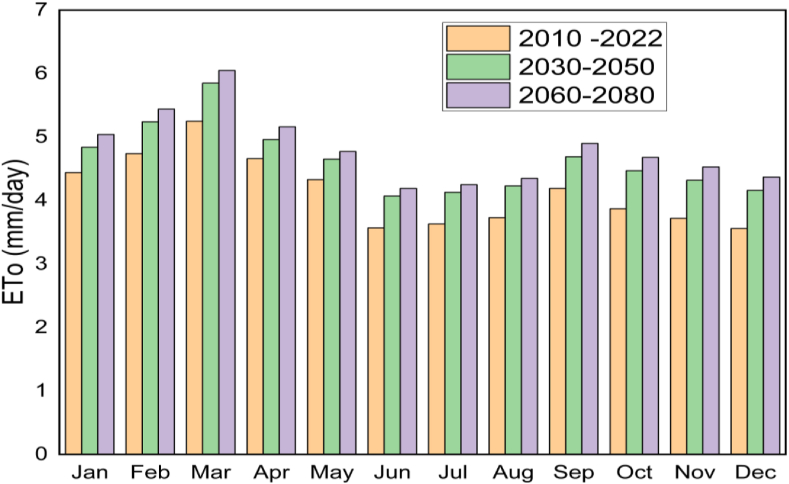
Reference evapotranspiration in the lower Kulfo catchment under climate change scenario.

Based on climate data analysis; January to March and July to September were very dry seasons indicating irrigation must be important for this duration. The average reference evapotranspiration under 2010–2022 (reference period), 2030 to 2050, and 2060 to 2080 were 4.1 mm/day, 4.6 mm/day, and 4.8 mm/day, respectively (Fig. 5). When compared to the reference period, the average reference evapotranspiration from 2030 to 2050 and 2060 to 2080 increased by 11.9 % and 16.2 %, respectively. This result demonstrated that future climate change will significantly increase soil surface temperature and evaporation [43] that indicates considering effective climate smart e agriculture in future.

#### 3.3.3 On crop and irrigation water requirement

**Fig. 6:**
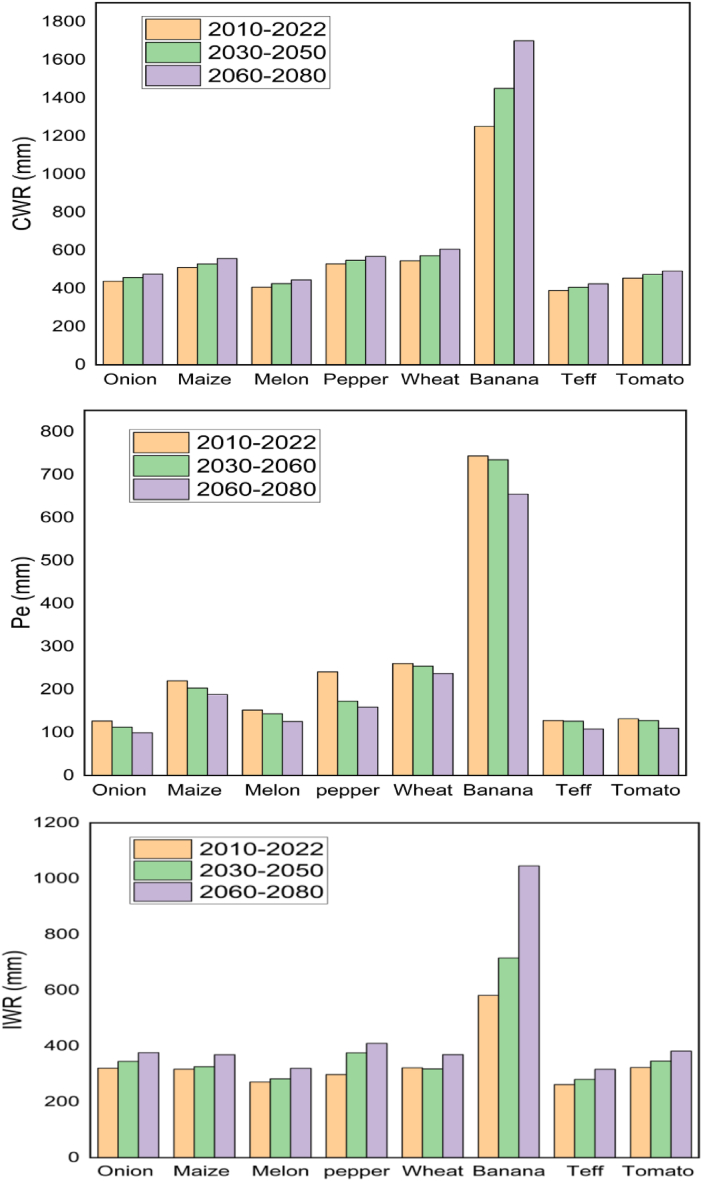
Crop water, effective rainfall, and irrigation under climate change scenarios.

The total seasonal crop water requirements for the dominant crop in the lower Kulfo Catchment were 4,529 mm, 4866.7 mm, and 5272.2 mm under the 2010 to 2022 (reference period), 2030 to 2050, and 2060 to 2080 climate change scenario, respectively (Fig. 6). In 2030 to 2050 and 2060 to 2080 climate change scenarios; the total crop water requirement was increased by 7.5 % and 16.4 % compared with reference period, respectively. Similarly, the total irrigation water requirement in 2030–2050 and 2060 to 2080 increased by 10.9 % and 33.11 %, respectively. This increasing trend of crop water requirement under climate change scenario may be due to a rise in temperature due to climate change and decreasing of available rainfall [44]. Total seasonal rainfall was decreased by 6.4 % (2030–2050) 16.1 % (2060–2080) compared with the reference period. Average crop water requirement of onion up to 440 mm per season and the estimated value of current study was less with this suggested value. The average seasonal crop water requirement of tef in Rift Valley Ethiopia ranged between 346.0 and 378.4 mm [28] and the value current study is also less than this indicated range. Based on [45], the impacts of climate change will increase crop water requirements by 40 % which indicates proper environment management is the main solution to conserve available water in the area. Irrigation water requirement is greatly influenced by rising temperatures, evaporation, and climate change may also have an impact on future water supplies globally [46]. The adaptation of technologies like the climate-smart agriculture concept needs measures and policies to reduce vulnerability and increase the capacity of producers, especially smallholders for effective climate change adaptation [47].

Where; CWR, Pe, and IWR were crop water requirement, effective precipitation, and irrigation water requirement, respectively.

#### 3.3.4 On irrigation interval

The average long and short irrigation intervals were found under banana and teff crops, indicating that teff crops were very sensitive to water stress. The average irrigation interval in 2010–2022, 2030 to 2050, and 2060 to 2080 climate change scenarios were 8 days, 7 days, and 5 days, respectively (Table 5), and the mean irrigation interval was decreased by 14 % (2030–2050), and 34 % (2060–2080) compared with reference period. Estimated value of irrigation interval for some crops was different because of length growing stage, root depth, and depletion factor of each crop are different. The mean value of irrigation interval for maize, onion and pepper under full irrigation practice were 7 days [48], 5 days [49], and 6 days [50], respectively and only maize irrigation interval was similar with value of the current study (Table 5). The remaining irrigation interval of crop was less than value of current study that shown irrigation interval for the same crop will vary from one place to place which may be due climate, soil and field management vartion. The average irrigation interval was decreased with the climate change scenario that was due to the increasing daily actual evapotranspiration rate. Sustainable water management practices and precision irrigation technologies play an important role in optimizing irrigation intervals and ensuring efficient water use, especially in the context of changing climatic conditions [46].

**Table 5:** Average irrigation interval under climate change scenario (days).

Crop type	Climate change scenario		
	2010 to 2022	2030 to 2050	2060 to 2080
Onion	6	5	4
Maize	7	6	4
Watermelon	7	6	5
Pepper	10	8	6
Wheat	7	6	5
Banana	16	13	9
Teff	5	5	4
Tomato	8	7	5
**Average**	**8**	**7**	**5**

In 2030 to 2050 and 2026 to 2080 climate change scenarios, the required irrigation water at the main canal was increased by 6.8 % and 18 %, respectively (Table 6). These projections highlight the potential implications of climate change on the availability of irrigation water in the Lower Kulfo Catchment, emphasizing the need for sustainable water management strategies to address evolving climatic conditions. The nimum and maximum probale irrigation flow was observed under April and March, respectively under all climate change scenarios.

**Table 6:** Monthly required irrigation water in the lower Kulfo catchment under climate change scenario (l/s).

Scenarios	Month (Dry season)			
	Jan	Feb	Mar	Apr
2010 to 2022	209.1	418.1	522.7	70
2030 to 2050	221.6	447.4	564.5	74.1
2060 to 2080	246.7	489.2	632.5	81.1

### 3.4 Allocation of irrigated land

#### 3.4.1 Developed optimization function

The average overall total production, production cost, and net benefit in the lower Kulfo catchment were estimated from the household interview and that was used as input to develop the objective function of the cropland allocation as presented in Table 7. The minimum and maximum net benefit in lower Kulfo catchment were observed under banana and tomato, respectively and the existing average net benefit of crop in the study area was 92,286.6 Ethiopian Birr per hectare (ETB/ha).

**Table 7:** Average overall total production, production cost, and net benefit.

Crop type	Production (tons/ha)	Total production (ETB/ha)	production cost (ETB/ha)	Net benefit (ETB/ha)
Onion	5.0	150000.0	40250.0	109,750.0
Maize	3.85	126885.0	25444.5	101,440.5
Watermelon	3.80	152000.0	50400.0	101,600.0
Pepper	5.15	221450.0	116945.0	104,505.0
Wheat	3.12	87360.0	23940.0	63,420.0
Banana	5.15	103000.0	48398.9	54,601.1
Teff	1.53	73246.4	10763.5	62,482.9
Tomato	6.41	192166.7	51673.0	140,493.7
**Average**	**4.3**	**138,263.5**	**45,976.9**	**92,286.6**

Land productivity of onion and tomato in the Rift Valley Lake basin was 8.85ton/ha and 7.45ton/ha, respectively [51]. The average land productivity of maize, wheat, and tomato in the Arba Minch climate zone were 6.57ton/ha [52], 6.03ton/ha [53], and 8.10ton/ha [54], respectively. Average land productivity of onion, pepper, and banana in Mirab abaya of the Gamo zone also 10.9ton/ha [54], 6.25ton/ha [55], and 27.4ton/ha [56], respectively. The mean land productivity of teff in the Rift Valley and watermelon in Koga were 3ton/ha [57], and 36ton/ha [58], respectievly. However, the land productivity of crops in the lower Kulfo catchment was too low compared with earlier estimated value, and the conducted research was due to a lack of good agricultural input and land management practices as observed during the field survey. The net benefit of onion production in Arba Minch Zuria woreda, and Hormat-Golina under good land management and full level of water application were 288,458 ETB/ha and 198,470 ETB/ha but net benefit onion of small hold farmer in lower Kulfo catchment was 74,750 ETB/ha [59] and value of current study was 109,750ETB/ha. The net benefit of maize, tomato, pepper, and teff in Hormat-Golina it located in the northern part of Ethiopia were 40,936ETB/ha, 474,105ETB/ha, 30,351ETB/ha and 27,057ETB/ha, respectively [60]. The net benefit of above crops except tomato in lower kulfo catchment were too high compared with Hormat-Golina. Those all above results and earlier study show that net production of crop was varied place to place due to adoption of agricultural technology and available of irrigation or rainfall. The objective function was developed by using the net benefit of the crop as multiplier coefficients with variables of allocated area subjected with four active constraints as shown below Eq (14)).

(14) $$\text{Maximaze}\;Z=109750X_{1}+101440.5X_{2}+101600.0X_{3}+104505X_{4}+63420X_{5}+54601X_{6}+62482.7X_{7}+140493.7X_{8}$$

Subject to.

A. Land constraint

The total land area occupied by each crop should not exceed the overall available land (Eq (15)).

(15) $$X_{1}+X_{2}+X_{3}+X_{4}+X_{5}+X_{6}+X_{7}+X_{8}\leq 1175.82$$

B. Water availability constraint

The minimum flow of Kulfo River was recorded in January that was 2.35 m^3^/s and based on [61], the minimum flow of river that consider for down stream user/ecology is 30 %. As result, only 1.65 m^3^/s of the River flow will be delieverd to the Lower Kulfo Cachment. Therefore, the minimum volume of available water was evaluated multiple deliver flow by duration of active crop growing period under worst dry season that was only four months. After 120-day crop start harvest and no more irrigation to be applied. Irrigation continues only for banana crop and irrigation water after 120 days of the banana was not include in optimization constraint. The estimated minimum volume of available irrigation water from the sources was 1705.54ha-m (Eq (16)).

(16) $$0.458X_{1}+0.453X_{2}+0.388X_{3}+0.426X_{4}+0.460X_{5}+0.831X_{6}+0.274X_{7}+0.503X_{8}\leq 1705.54$$

C. Expected total yield constraints

The main aims of this constraint improved grade up existing land productivity that describe in Table 7 to recommended maximum value. The average expected maximum land productivity of crops was summarized in Table 8 that was collected from irrigation and drainage paper manual number 33 (FAO 33).

**Table 8:** Expected land productivity (FAO 33) [62].

Crop type	Land productivity (ton/ha)	Crop type	Land productivity (ton/ha)
Onion	10.9	Wheat	6.03
Maize	6.57	Banana	27.4
watermelon	36.0	Teff	3.0
Pepper	6.25	Tomato	8.1
Average yield (ton/ha)	13.03 1175.80 15,321.38		
Total available area (ha)			
Total expected yield (ton)			

The average expected total production of eight dominant crops in the lower Kulfo catchment with the available area was 15,321.38ton (Table 8), and non-negativity constraints were discussed earlier for two optimization scenarios and

expected yield constraints was described in Eq (17).

(17) $$10.9X_{1}+6.57X_{2}+36X_{3}+6.25X_{4}+6.03X_{5}+27.4X_{6}+3X_{7}+8.13X_{8}\geq 15,321.38$$

D. Total production cost constraint

The average crop production cost of study area was 45,976.9ETB/ha and the total production cost of scheme also 54,060,514 ETB. Therefore, the total production cost of current study was less than or equal to actual estimated value (Eq (18)).

(18) $$40250X_{1}+25444.5X_{2}+50400X_{3}+116945X_{4}+23940X_{5}+48398.9X_{6}+10763.5X_{7}+51673X_{8}\leq 54060514$$

#### 3.4.2 GAMS code optimization output

The GAMS (General Algebraic Modeling System) code output suggests that tomato, maize and watermelon crops were the most beneficial or profitable crops for the Arba Minch area. This indicates that, in terms of net benefit, cultivating these crops provides the best returns and the code program required 6 iterations to find the optimal solution and took 0.06 s. The optimal irrigated land was allocated for tomato (60.4 %), maize (20.8) and watermelon (18.8 %) in scenario1 optimization and net benefit from this scenario was 1.47*108 Ethiopian Birr (Table 9). This result was shown tomato was highly profitable crop compared with maize and watermelon. The remaining five crops was considered non beneficiary in the study area based with constraints of problem.

**Table 9:** Output of GAMS code program.

Crop type	Scenario 1	Scenario 2
	Area (ha)	Area (ha)
Onion	0.0	0.0
Maize	244.6	564.9
Watermelon	221.1	239.8
Pepper	0.0	0.0
Wheat	0.0	0.0
Banana	0.0	0.0
Teff	0.0	0.0
Tomato	710.1	371.1
Net benefit (ETB)	1.47*E+8	1.34E+8

According to household interviews, smallholder farmers predominantly cultivate maize compared to other crops or fruits because it serves as the primary source of food security in the area. Under existing farmers adaptation practices, maize covers an average minimum area of 564.9ha (48 %). Consequently, optimization strategies were developed based on existing farmers' perceptions (scenario 2), prioritizing maize cultivation with an area equal to or greater than the minimum covered by maize in existing irrigation practices (X_2_). In scenario 2 optimization, the most optimal crops were identified as maize, tomato, and watermelon. The allocated areas for each crop under scenario 2 were 564.9ha (48 %) for maize, 371.1ha (31.6 %) for tomato, and 239.8ha (20.4 %) for watermelon. The allocation of land for maize cultivation by the GAMS code program closely similar with farmers' adaptation, suggesting the scheme's economic viability when a large area is devoted to maize cultivation. In scenario 2, the optimal net benefit per season was calculated to be 1.34*10^8^ Ethiopian Birr (ETB), as indicated in Table 9. This represented a decrease of 19.1 % compared to the net benefit in scenario 1, emphasizing the trade-off between maximizing profit and ensuring food security. As a result, smallholder farmers to be grow maize, tomato, and watermelon using both irrigation and rainfed agriculture. The implementation of postharvest handling practices, such as harvesting, precooling, cleaning or packaging, storage, and transportation, significantly contributes to preserving quality of tomato and watermelon fruits after harvest [63]. Also, the use of appropriate postharvest treatment methods like refrigeration, postharvest heat treatment, modified atmosphere packaging (MAP), and 1-methylcyclopropene (1-MCP) and calcium chloride (CaCl_2_) application is important [64].

#### 3.4.3 Land use plan for optimal crop

The duration of the initial, development, mid, and late stages of crop growth to formulating a land use plan and to determining irrigation requirement of crop [65]. Due to that, the growth stage of the current identified crop was collected from the irrigation and drainage paper manual number 56 (FAO 56) as described in Table 10.

**Table 10:** Growth stage of selected crop ([27].

Crop/Stage	Initial stage	Development stage	Mid-stage	Late	Total
Maize	20	35	40	30	125
Watermelon	20	30	30	30	110
Tomato	30	40	40	25	135

Offseason represents duration of land preparation for the next irrigation season and maize, watermelon, and tomato are scheduled to be harvested on May 5, April 20, and May 15, respectively (Table 11). The beginning of the second growing season is expected in July, following land preparation in June, and this calendar plan was developed based total growing period of optimal crop as described in Table 10.

**Table 11:** plans of irrigation calendar, offseason (land preparation time), and monthly irrigation required.

Crop/Month	Dec	Jan	Feb	Mar	Apr	May	Jun	Jul	Aug	Sep	Oct	Nov
Maize	Offseason					5	Offseason					5
Watermelon	Offseason				20		Offseason				20	
Tomato	Offseason					15	Offseason					15

## 4 Conclusion and recommendation

Future projections indicate rising mean monthly temperatures and reduced rainfall, particularly in August (2030–2050) and March (2060–2080). The average reference evapotranspiration, crop water and irrigation requirement of study was increased under 2030 to 2050 and 2060 to 2080 climate change scenarios compared with reference period (2010–2022). Declining of irrigation intervals under climate change scenarios (2030–2050, and 2060–2080) shows increasing of daily actual evapotranspiration rates. The most economical crop in the study area under all optimization scenarios were tomato, watermelon and maize. While these crops yield high profits, maize remains important for food security, necessitating a balance between profitability and food that indicates optimal crop allocations was considering both economic benefits and food security concerns in agricultural decision-making. There are two crop planting frequency in the area that was from January to May and from July to November. December and June are dedicated to land preparation for the first and second growing seasons, respectively. Generally, this research provides useful guidance to policymakers on how to implement and embrace climate change resilience agriculture to ensure sustainable food security in the future. Furthermore, this study will be used to improve small-scale farmers' awareness of irrigation scheduling and crop selection in the area through workshops and training. Future researchers will assess the effects of post-harvesting technologies on the quality and storage duration of watermelon and tomato fruits.

## Data availability statement

Data will be made available on request.

## Additional information

No additional information is available for this paper.

## CRediT authorship contribution statement

**Birara Gebeyhu Reta:** Formal analysis, Data curation, Conceptualization. **Samuel Dagalo Hatiye:** Methodology, Formal analysis. **Mekuanent Muluneh Finsa:** Conceptualization.

## Declaration of competing interest

The authors declare that they have no known competing financial interests or personal relationships that could have appeared to influence the work reported in this paper.

## References

[1] Tesema T., Gebissa B. (2022). Multiple agricultural production efficiency in horro district of horro guduru wollega zone, western Ethiopia, using hierarchical-based cluster data envelopment analysis. Sci. World J..

[2] Zhang J. (2021). Challenges and opportunities in precision irrigation decision-support systems for center pivots. Environ. Res. Lett..

[3] Létourneau G., Caron J. (2019). Irrigation management scale and water application method to improve yield and water productivity of field-grown strawberries. Agronomy.

[4] Adamtie Temesgen F., selie Abeba H., Mitku Demeke T. (2022). Crop water requirement and irrigation scheduling of dry season irrigated crops in Pawe district, lowland hot humid area of Ethiopia. Int. J. Sch. Res. Life Sci..

[5] Mirzaei A., Azarm H., Naghavi S. (2022). Optimization of cropping pattern under seasonal fluctuations of surface water using multistage stochastic programming. Water Supply.

[6] Boatemaa A., Incoom M., Kwadwo E., Odai S.N. (2022). Impacts of climate change on crop and irrigation water requirement in the Savannah regions of Ghana. J. Water Clim. Chang..

[7] Soares D., Paço T.A., Rolim J. (2023). Assessing climate change impacts on irrigation water requirements under mediterranean conditions—a review of the methodological approaches focusing on maize crop. Agronomy.

[8] Tian X., Dong J., Jin S., He H., Yin H., Chen X. (2023). Climate change impacts on regional agricultural irrigation water use in semi-arid environments. Agric. Water Manag..

[9] Oyelakin (2024). Analysing urban flooding risk with CMIP5 and CMIP6 climate projections. Water.

[10] Feyissa T.A., Demissie T.A., Saathoff F. (2023). Evaluation of general circulation models CMIP6 performance and future climate change over the omo river basin , Ethiopia. Sustainability.

[11] Sen B. (2023). Determining the changing irrigation demands of maize production in the cukurova plain under climate change scenarios with the CROPWAT model. Water.

[12] Yeboah K.A., Akpoti K., Kabo-bah A.T., Ofosu E.A., Siabi E.K. (2022). Assessing climate change projections in the Volta Basin using the CORDEX- Africa climate simulations and statistical bias-correction Assessing climate change projections in the Volta Basin using the CORDEX-Africa climate simulations and statistical bias-correction. Environ. Challenges.

[13] Betele D., Gebul M.A., Andries J., Plessis D. (2023). Assessment of irrigation water allocation. Koftu , Ethiopia.

[14] Pal J.S. (2007). Regional climate modeling for the developing world: the ICTP RegCM3 and RegCNET. Bull. Am. Meteorol. Soc..

[15] Zenis F.M., Supian S., Lesmana E. (2018). Optimization of land use of agricultural farms in Sumedang regency by using linear programming models. IOP Conf. Ser. Mater. Sci. Eng..

[16] Sofi N.A., Ahmed A., Ahmad M., Bhat B.A. (2015). Decision making in agriculture: a linear programming approach. Int. J. Mod. Math. Sci. J. homepage www.ModernScientificPress.com.

[17] Nimah M.N., Bsaibes A., Alkahl F., Darwish M.R., Bashour I. (2003). Optimizing cropping pattern to maximize water productivity. River Basin Manag. Ii.

[18] Hao L., Su X., Singh V.P. (2018). Cropping pattern optimization considering uncertainty of water availability and water saving potential. Int. J. Agric. Biol. Eng..

[19] Jayne T.S., Chamberlin J., Headey D.D. (2014). Land pressures, the evolution of farming systems, and development strategies in Africa: a synthesis. Food Pol..

[20] Demmissie N.G., Demissie T.A., Tufa F.G. (2018). Predicting the impact of climate change on Kulfo River flow.

[21] Reta B.G., Hatiye S.D., Finsa M.M. (2024). Assessment of irrigation water management performance indicators and mitigation measure in Arba minch irrigation. Adv. Agric..

[22] Yubing Fan S.C.P., R. M. (2018). Multi-crop production decisions and economic irrigation water use efficiency : the effects of water climatic determinants. Water.

[23] Wright A., Hudson D., Mutuc M. (2013).

[24] Goebel T.S., Lascano R.J., Acosta-Martinez V. (2016). Evaluation of stable isotopes of water to determine rainwater infiltration in soils under conservation reserve program. J. Agric. Chem. Environ..

[25] Chen C., Hsu H., Liang H. (2021). Evaluation and comparison of CMIP6 and CMIP5 model performance in simulating the seasonal extreme precipitation in the Western North Pacific and East Asia. Weather Clim. Extrem..

[26] Leander R., Buishand T.A. (2007). Resampling of regional climate model output for the simulation of extreme river flows. J. Hydrol..

[27] Allen R.G., Pereira L.S., Raes D., Smith M. (1998).

[28] Hordofa T. (2020). Crop water requirement and crop coefficient of tef (eragrostis tef) in central Rift Valley of Ethiopia.

[29] Hooper B.P. (2003). Integrated water resources management and river basin governance. Water Resour..

[30] Bowen R.L., Young R.A. (1985). Financial and economic irrigation net benefit functions for Egypt's northern delta. Water Resour. Res..

[31] Twum E.K.A., Nii-Annang S. (2015). Impact of soil compaction on bulk density and root biomass of quercus petraea L. At reclaimed post-lignite mining site in lusatia, Germany. Appl. Environ. Soil Sci..

[32] Bharati L. (2008). Integration of economic and hydrologic models: exploring conjunctive irrigation water use strategies in the Volta Basin. Agric. Water Manag..

[33] Gülser C., Ekberli I., Candemir F., Demir Z. (December 2018, 2012). Spatial Variability of Soil Physical Properties in a Cultivated Field.

[34] Habtewold B.M., Gelu G. (2020). At Arbaminch Zuria District in SNNPR , Ethiopia EvaluationofIrrigationRegimeforOnionAlliumCepaLAtArbaminchZuriaDistrictinSNNPREthiopia.

[35] Kedir Y. (2015). Estimation of conveyance losses of wonji-shoa sugarcane irrigation scheme , Ethiopia.

[36] Adnan M.S., Aliff M., Anuar M., Nda M. (2017).

[37] Deboer B.D.W., Chu S.T., Unoff R. (2001).

[38] Zerssa G., Feyssa D., Kim D., Eichler-löbermann B. (2021). Challenges of smallholder farming in Ethiopia and opportunities by adopting climate-smart agriculture. Agriculture.

[39] Malhi G.S., Kaur M., Kaushik P. (2021). Impact of climate change on agriculture and its mitigation strategies : a review. Sustainability.

[40] Gebeyhu B., Markos G. (2023). Assessment of soil mulching field management , and deficit irrigation effect on productivity of watermelon varieties , and AquaCrop model validation. Heliyon.

[41] Setu T., Legese T., Teklie G., Gebeyhu B. (2023). Effect of furrow irrigation systems and irrigation levels on maize agronomy and water use efficiency in Arba Minch. Heliyon.

[42] Sinore T., Wang F. (2024). Impact of climate change on agriculture and adaptation strategies in Ethiopia : a meta-analysis. Heliyon.

[43] Boatemaa A., Incoom M. (2022). Impacts of climate change on crop and irrigation water requirement in the Savannah regions of Ghana Impacts of climate change on crop and irrigation water requirement in the Savannah regions of Ghana. J. Water Clim. Chang..

[44] S. A. Salman, S. Shahid, H. A. Afan, M. S. Shiru, N. Al-ansari, and Z. M. Yaseen, “Changes in climatic water availability and crop water demand for Iraq region,” Sustainability, vol. 12, no. 8, pp. 14–27.

[45] Gunther (2007). Climate change impacts on irrigation water requirements : effects of mitigation , 1990-2080. Technol Forecast Soc Chang Climate change impacts on irrigation water requirements.

[46] Nikolaou G., Neocleous D., Christou A., Kitta E. (2020). Implementing sustainable irrigation in water-scarce regions under the impact of climate change. Agronomy.

[47] Azadi H., Burkart S., Moghaddam S.M., Goli I. (2022). Climate smart agriculture : mitigation and adaptation strategies at the global scale climate smart agriculture : mitigation and adaptation strategies at the global scale. Clim. Innov..

[48] Science E. (2021).

[49] Ayza A. (2018). Development of optimum irrigation regime for onion production at Arba minch. Southern Ethiopia.

[50] Adeoye P.A., Adesiji R.A., Oloruntade A.J., Njemanze C.F. (2014). Effect of irrigation intervals on growth and yield of bell pepper (Capsicum annuum) in a tropical semi-arid region.

[51] Nishikawa Y. (2018). Economic feasibility of small-scale onion and tomato production in the central Rift Valley in Ethiopia: evidence from adama and doddota districts. Polit. Sociol. Japanese Pacifism.

[52] Yemane G., Mekonen A., Kassa T. (2015). Field experimentation based simulation of yield response of maize crop to deficit irrigation using AquaCrop model, Arba Minch, Ethiopia. Afr. J. Agric. Res..

[53] Khan A.Q., Robe B.L., Girma A. (2020). Evaluation of wheat genotypes (Triticum aestivum L.) for yield and yield characteristics under low land area at Arba Minch, Southern Ethiopia. African J. Plant.

[54] Fikre G., Mensa A., Wodaje A. (2022). Adaptability evaluation of improved Tomato (Lycopersicon esculentum Mill.) varieties for yield and other quantitative traits in Arba Minch, Southern Ethiopia. Int. J. Agric. Res. Innovat. Technol..

[55] Alemu A., Wodajo A., Chuntal K. (2016). Performance evaluation of elite hot pepper(Capsicum annum) varieties for yield and yield components at derashea, south-eastern Ethiopia. Int. J. Res..

[56] Zenebe M., Poesen a, Nyssen J., Verstraeten J., Govers G., Deckers G. (2008). Magnitude and dynamics of runoff and sediment transport in the geba river. Assessment.

[57] Mengiste Yenesew (2015).

[58] Enyew A., Tewabe D., Tsige A. (2020). Determining the irrigation regime of watermelon at Koga and Rib irrigation schemes in Amhara Region, Ethiopia. Cogent Food Agric..

[59] Tadesse T., Sharma P.D., Ayele T. (2022). Effect of the irrigation interval and nitrogen rate on yield and yield components of onion (Allium cepa L.) at Arba minch, southern Ethiopia. Adv. Agric..

[60] Kerebih M.S. (2021).

[61] Chen H., Teegavarapu R. (December 2019, 2020). Comparative Analysis of Four Baseflow Separation Methods in the South Atlantic-Gulf Region of the U . S.

[62] Doorenbos J., Kassam A.H. (1986).

[63] Pokhrel B. (2021). Review on post-harvest handling to reduce loss of fruits and vegetables Review on post-harvest handling to reduce loss of fruits and vegetables. Int. J. Hortic. Food Sci.

[64] Arah I.K., Ahorbo G.K., Anku E.K., Kumah E.K., Amaglo H. (2016). Postharvest handling practices and treatment methods for tomato handlers in developing countries : a mini review. Adv. Agric..

[65] Antonio Cano A.D., J. M. (2022). José jesús pardo, “determining irrigation requirements of extensive crops using the typical meteorological year adjusted to the growing,”. Agronomy.

